# Effects of rowing stroke rates on lower extremity intra-joint coordination variability in experienced young rowers

**DOI:** 10.1371/journal.pone.0286999

**Published:** 2023-12-21

**Authors:** Faezeh Pakravan, Ali Abbasi, Zahra Noorinezhad, Zdenek Svoboda, Mehdi Khaleghi Tazji, Siavash Dastmanesh

**Affiliations:** 1 Department of Biomechanics and Sports Injuries, Faculty of Physical Education and Sports Sciences, Kharazmi University, Tehran, Iran; 2 Department of Sport Sciences, Faculty of Education and Psychology, Shiraz University, Shiraz, Iran; 3 Department of Sport Science, Payame Noor University (PNU), Tehran, Iran; 4 Faculty of Physical Culture, Palacky University Olomouc, Olomouc, Czech Republic; 5 Department of Sport Sciences, Abadeh Branch, Islamic Azad University, Abadeh, Iran; Ningbo University, CHINA

## Abstract

The purpose of this study was to examine the effects of rowing stroke rates on lower extremity intra-joint coordination variability in professional rowers. Fifteen experienced young rowers volunteered to participate in this study. Kinematic data were recorded at different rowing speeds with seven Vicon cameras. The continuous relative phase (CRP) and CRP variability (CRPV) were used to calculate joint coordination and coordination variability, respectively, for the hip, knee, and ankle in the sagittal and horizontal planes, and a comparison was made among different rowing stroke rates. A vector analysis repeated measure ANOVA using statistical parametric mapping revealed that there were statistically significant differences in the hip–ankle, hip–knee, and knee–ankle CRPs for rowing at different stroke rates. Moreover, there was higher CRPV in the mid-drive and mid-recovery phases and less variability in the transition from the drive phase to the recovery phase. The results demonstrate the importance of knee joint in rowing tasks in experienced rowers during submaximal rowing stroke rate and the shift of movement to the hip at higher rowing stroke rate. Moreover, there was a smaller variability during drive-to-recovery transition, which may suggests an increased risk for overuse injuries.

## Introduction

Rowing is a cyclical movement like cycling and running that is a professional sport in the Olympic Games. It is also an athletic exercise that is done on ergometers in the gym to improve physical fitness. This activity is considered to be a safe and low-impact sport with little risk of severe injuries; however, skilled rowers are known to suffer from overuse injuries. The most common injuries in rowing are related to overuse injuries that is caused by the repetitive nature of this athletic task [1–5]. It accounts for 72.1% and 69.5% of all rowing-related injuries for females and males, respectively [1, 6, 7]. Previous studies have linked rowing injuries with intensity or volume of training, poor technique, fatigue, overload, and rapid changes in training frequency [1, 3, 4, 8]. The two most common sites of rower injury are the lower back and the knee [9], with knee injuries representing 15.91% of total injuries [10]. Patellofemoral pain syndrome, tendinopathy, and iliotibial band friction syndrome are some examples of knee injuries in rowing [1, 8]. As the rowing motion requires the knee to move through its full range of motion (ROM) [1], some knee injuries might be due to the repetition of the flexion and extension motions under load [4, 11, 12]. In addition, Rachnavy (2012) found that the rowers with higher hip and knee ROM in sagittal plane during rowing significantly had more knee injury compared with the rowers with lower hip and knee ROM [3].

Rowing strokes can change from low to high intensity during training and competition to achieve optimal levels of performance and technique. Redgrave (1995) and Nolte (2005) mentioned critical biomechanical parameters in ergometer rowing techniques such as stroke length, duration and ratio of the stroke phases, forces of the stroke on the handle and foot stretcher, stroke power, trajectory of the handle motion, body posture, and body joint loads. Some of these parameters are dependent on stroke rate [13, 14]. The identification of changes in joint and segment kinematics at different rowing intensities not only helps coaches to identify the proper joints responsible for increasing intensity but also can help to identify possible reasons for injury mechanisms and changes in technique. Previous studies reported an increase in femoral and lumbo-pelvic ROM during rowing at higher intensities and linked this ROM increasing with injuries [15, 16]. However, in these studies a single joint kinematics parameter (ROM) was examined during rowing, while nonlinear dynamics analysis methods such as the coordination pattern of joints or segments have been suggested to provide a better understanding of the biomechanics of movement [17–21]. Quantifying kinematic coordination is a tool used to determine coupled movements between two segments, and coordination variability (CRPV) quantifies the variety of movement patterns that an individual uses during a task and can provide a measure of the flexibility and adaptability of an individual’s motor system [22, 23]. Moreover, researchers have connected lower CRPV with pathological conditions when compared to age- or activity-matched individuals without the pathology [24]. Because overuse injuries can arise during repetitive movement like rowing, the change in rowing speed can change the load demand on the joints and segments and CRP variability may reduce at higher speed of rowing and increase the risk for overuse injuries on the lower extremity [25].

There are limited studies on lower extremity coordination and variability during rowing at different intensities. However, McGregor et al. (2004) investigated spinal and pelvic motion and the force generated at the handle at three different stroke rates. They showed that there is no change in the magnitude peak of the spinal torque generated during different stroke rates, but a shift in the instant of occurrence was noticed. They also found changes in pelvic rotation at the catch and finish of the stroke [16]. Potvin-Gilbert (2018) found that rowers have a reduction in knee angle variability on the water and high intensity compared to rowing on the ergometer, which can be due to increased difficulty of the task. They also showed that increasing the intensity and distance of rowing does not affect the variability of the knee joint angle during ergometer rowing [26]. A review of the literature still does not reveal whether different rowing stroke rates can affect the coordination and coordination variability of segments. However, studies on coordination variability during running at different speeds have shown that coordination variability reduces with increase in running speed because of reduction in degree of freedom of movement at higher speed [25]. The purpose of this study was to examine the effects of rowing stroke rates on lower extremity inter-joint coordination and its variability in professional rowers. We hypothesized that 1) lower extremity inter-joint coordination patterns differ among different rowing stroke rates and 2) lower extremity inter-joint coordination variability decreases with an increase in rowing stroke rate.

## Methods

### Participants

Fifteen experienced rower girls without any injuries volunteered to participate in this study (they were members of the professional rowing clubs at Olomouc with more than 3 years rowing experience). Their age, rowing experience, height, and mass were 13.83 ± 1.19 years, 4.04 ± 0.89 years, 172.43 ± 8.04 cm, and 68.34 ± 13.07 kg, respectively. All participants were competitive athletes and were experienced in working on a Concept II rowing ergometer (Concept Inc., Morrisville, Vermont). The study was approved by the Ethics Committee of the Palacky University of Olomouc, and informed written consent was obtained from the participants before the experimental session. Rowers with a current episode of low back pain or any other serious musculoskeletal injury were excluded from participation in this study.

### Experimental design

A seven-camera motion capture system (Vicon MX, Vicon Motions Systems, Oxford, UK) was used to record kinematic data at a sampling rate of 200 Hz. A Concept II rowing ergometer was placed in the center of the room. The calibration was performed according to the manufacturer’s instructions. All participants changed into a spandex suit, which is a standard practice attire, and wore their personal training shoes during testing. A total of 16 markers were attached to their landmarks based on a full-body plug-in gait model [27]. Participants were asked to perform a brief warm-up on the rowing ergometer to accustom themselves to the equipment and practice rowing with the markers. After warm-up, the participant stood in an anatomical position to record the static test. They performed rowing test at speed 1 (rowing at 17–20 revolutions per minute [rpm]), speed 2 (rowing at 20–24 rpm), speed 3 (rowing at 24–28 rpm), and speed 4 (rowing at 28–36 rpm). They performed each rowing test for three minutes, with three minutes of rest between each interval, and kinematic data were recorded during each rowing test once the rate was maintained. Visual feedback of both the power output and stroke frequency was displayed on a monitor placed in front of them.

### Data processing

**Fig 1:**
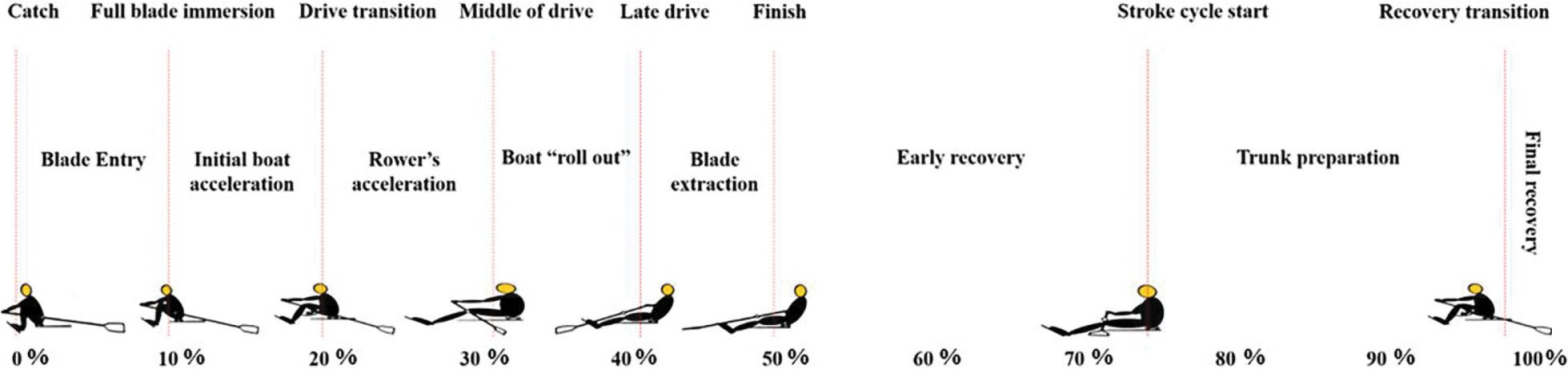
Positions and phases of rowing stroke based on Kleshnev’s description (Kleshnev 2016).

Raw marker trajectory data during rowing were filtered using a second-order, zero-lag Butterworth low-pass filter with an 8-Hz cutoff frequency. Markers were labeled and gap filled, and lower limb joint angles were calculated using Nexus 2.4.2 software (Vicon Motion Systems, Oxford, UK). Rowing cycles were obtained based on the trajectory of the horizontal position of the marker on the right wrist. Kinematic data of the middle seven rowing stroke sequences were selected for further analysis. The drive and recovery phases for each cycle were separately normalized to 50 points and time normalized to 100% of the rowing stroke; therefore, the first point of each normalized cycle was the catch position and the 50^th^ point was the finish position to allow for comparisons of rowing form among intensities (Fig 1). The kinematic data of dominant leg of participants were analyzed using MATLAB software (MATLAB, MathWorks, USA).

### Coordination and coordination variability calculation

Based on previous studies, the continuous relative phase (CRP) and CRP variability (CRPV) were used to calculate joint coordination and coordination variability, respectively [17, 19]. CRP and CRPV were calculated for five couplings of hip flexion/extension–ankle plantar/dorsi flexion (Hip FL/EX–ankle PF/DF), knee flexion/extension–ankle plantar/dorsi flexion (Knee FL/EX–ankle PF/DF), hip flexion/extension–knee flexion/extension (Hip FL/EX–Knee FL/EX), hip flexion/extension–knee internal/external rotation (Hip FL/EX–knee IR/ER), and knee internal/external rotation–ankle plantar/dorsi flexion (Knee IR/ER–ankle PF/DF). These joint couplings were selected based on the normal joints movement pattern during the close kinematic chain rowing task.

The minimum (min) and maximum (max) angles for the series of seven rowing cycles in each condition were used to normalize the angular position (*θ*) for each of the 100 data points (*i*). The angular velocity (*ω*) data were normalized to the maximal (max|*ω*|) velocity within the seven rowing cycles. A phase plane for each joint throughout a rowing cycle was constructed by plotting normalized angular positions (*θ*: *x*-axis) versus normalized angular velocities (*ω*: *y*-axis). The phase angle (*Ф*) was calculated as *Ф*_*A*_ = tan^−1^ (*ω*/*θ*) along each data point of the rowing cycle. Normalized angular position (*θ*_*inorm*_) and angular velocity (*ω*_*inorm*_) profiles were calculated by using the following equations, respectively:

$$\theta_{inorm}=\frac{2[\theta_{i}-\min(\theta_{i})]}{\max(\theta_{i})-\min(\theta_{i})}-1,$$ (1)

$$\omega_{inorm}=\frac{\omega_{i}}{\max|\omega_{i}|},$$ (2)

where *i* is each data point of the rowing cycle.

For each of the oscillators, the phase angle (*Ф*) was obtained by calculating the four-quadrant arctangent angle relative to the right horizontal axis at each instant in the kicking cycle [17]. CRP was calculated by subtracting the phase angle of the proximal joint from that of the distal joint and used as the coordination of two joints according to

$$\mathrm{CRP}(i)=\Phi_{A}(i)–\Phi_{B}(i)$$ (3)

where *Ф*_*A*_(*i*) and *Ф*_*B*_ (*i*) are the phase angle of distal and proximal oscillators, respectively, at data point *i* in the rowing cycle. CRP values can range from 0° to 180° and 0° to −180°: in-phase (0° ≤ CRP ≤ ±30°), when two joints move in the same direction; antiphase (±150° ≤ CRP ≤ ±180°), when two joints move in the opposite direction; and out of phase (±30° ≤ CRP ≤ ±150°). Positive relative phase values indicate that the distal joint is ahead of the proximal segment in phase space, and negative relative phase values indicate that the proximal joint is ahead in phase space. Positive and negative slopes indicate that the distal and proximal joint is moving faster in phase space, respectively [28].

### Statistical analysis

A statistical parametric mapping (SPM) repeated measures analysis of variance and paired sample t-test as post-hoc tests were used to detect significant differences among CRP and CRPV waveforms (v.M0.1, www.spm1d.org). Statistical significance for repeated measure ANOVA was set at *α* = 0.05 and it was adjusted for post-hoc tests by dividing 0.05 by the number of comparisons. All statistical analyses were conducted in MATLAB software.

## Results

**Fig 2:**
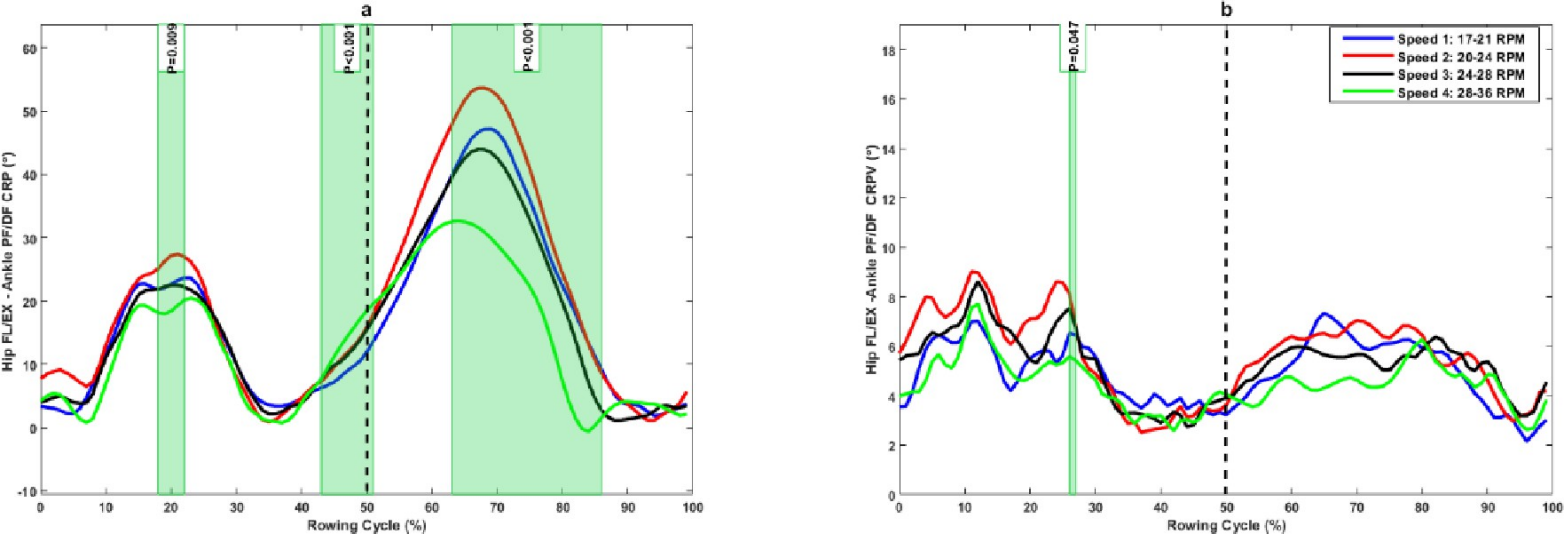
(a) Hip FL/EX–ankle PF/DF continuous relative phase and (b) its variability at different rowing speeds. The green shaded portions illustrate times in the stroke cycle when there was a significant difference between stroke rates.

The results showed that hip FL/EX–ankle PF/DF coupling was significantly greater in-phase at mid-drive phase (18%–21%) and significantly less in-phase at late drive phase (42%–51%) at higher rowing speed compared to slower rowing speed. It was significantly more in-phase at mid-recovery phase (62%–87%) at higher rowing speed compared to lower rowing speed (Fig 2A). Moreover, rowers showed a significantly lower hip FL/EX–ankle PF/DF CRPV just in the mid-drive phase (27%–28%) at higher rowing speed compared to lower rowing speed (Fig 2B).

**Fig 3:**
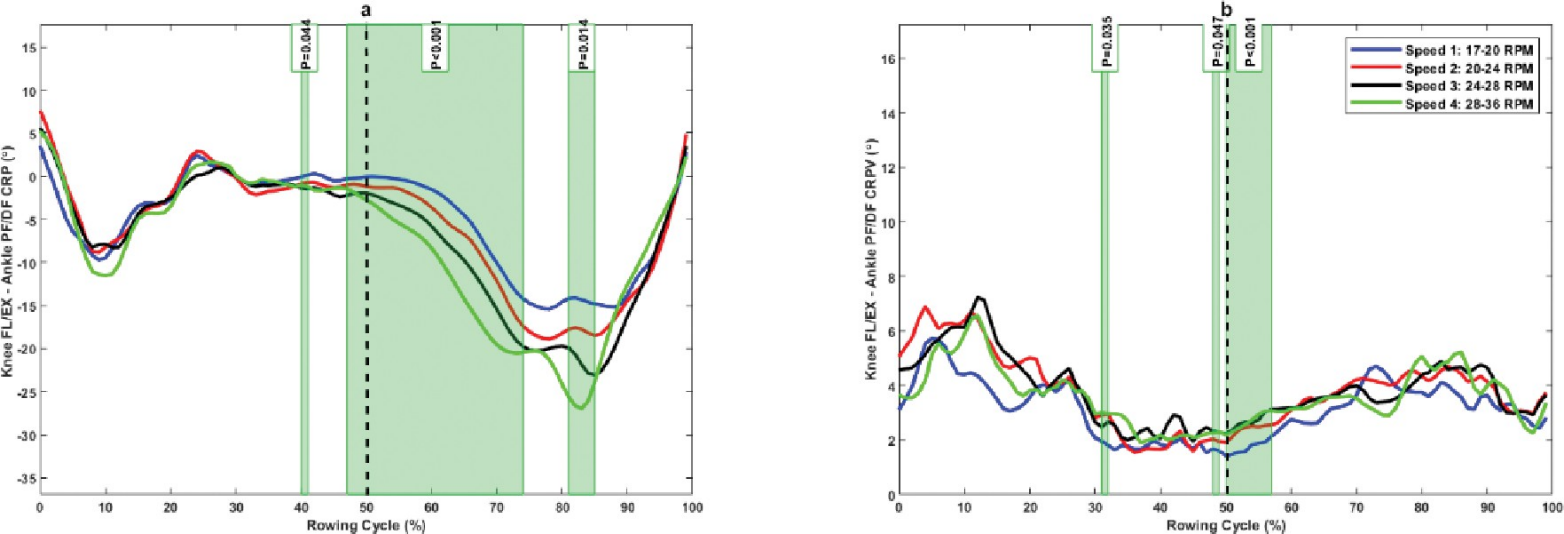
(a) Knee FL/EX–ankle PF/DF continuous relative phase and (b) its variability at different rowing speeds. The green shaded portions illustrate times in the stroke cycle when there was a significant difference between stroke rates.

The knee FL/EX–ankle PF/DF CRP was significantly more in-phase in the late drive phase (40%–41% and 47%–50%), early to mid-recovery phase (50%–74%), and in the mid-recovery phase (81%–85%) at slower rowing speed compared to higher rowing speed (Fig 3A). Moreover, rowers showed a significantly lower knee FL/EX–ankle PF/DF CRPV in the mid-drive phase (32%–33%), late drive phase (48%–49%), and early recovery phase (50%–57%) at higher rowing speed compared to lower rowing speed (Fig 3B).

**Fig 4:**
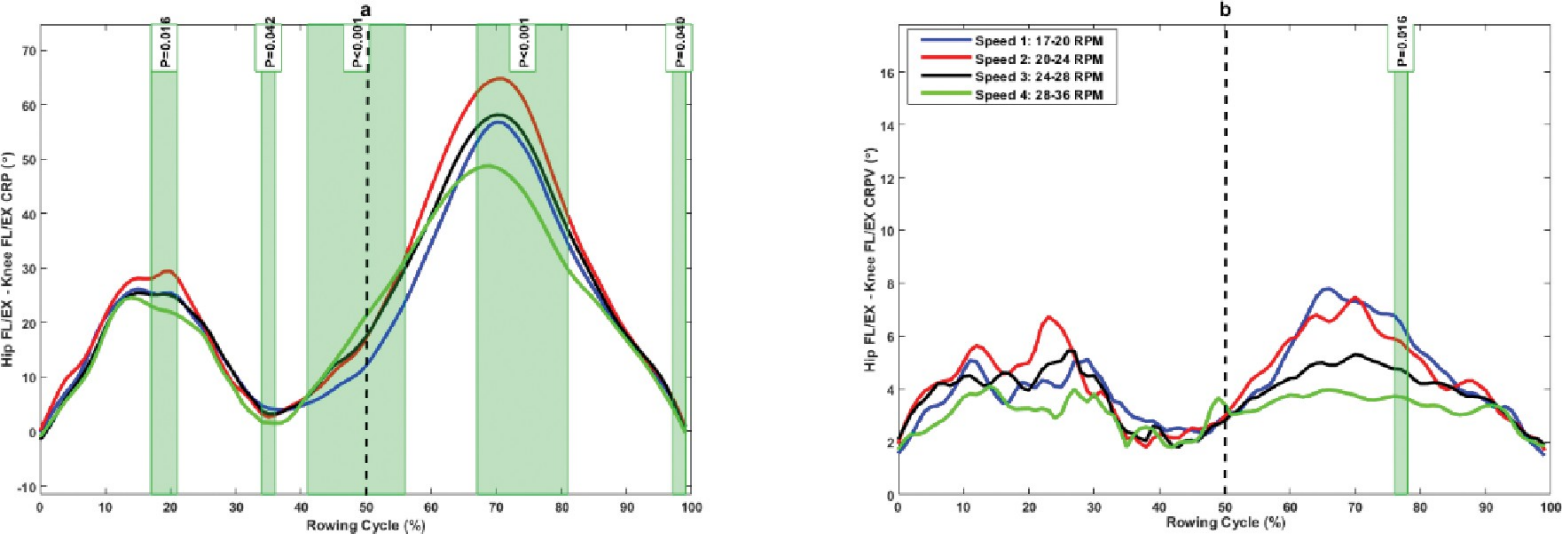
(a) Hip FL/EX–knee FL/EX continuous relative phase and (b) its variability at different rowing speeds. The green shaded portions illustrate times in the stroke cycle when there was a significant difference between stroke rates.

The hip FL/EX–knee FL/EX CRP was significantly more in-phase in the mid-drive phase (17%–21%), late drive phase (33%–35%), mid-recovery phase (67%–81%), and late recovery phase (97%–99%), but significantly less in-phase in the late drive phase (41%–50%) and early recovery phase (50%–56%) at higher rowing speed compared to lower rowing speed (Fig 4A). Moreover, rowers showed a significantly lower hip FL/EX–knee FL/EX CRPV in the late recovery phase (75%–77%) at higher rowing speed compared to lower rowing speed (Fig 4B).

**Fig 5:**
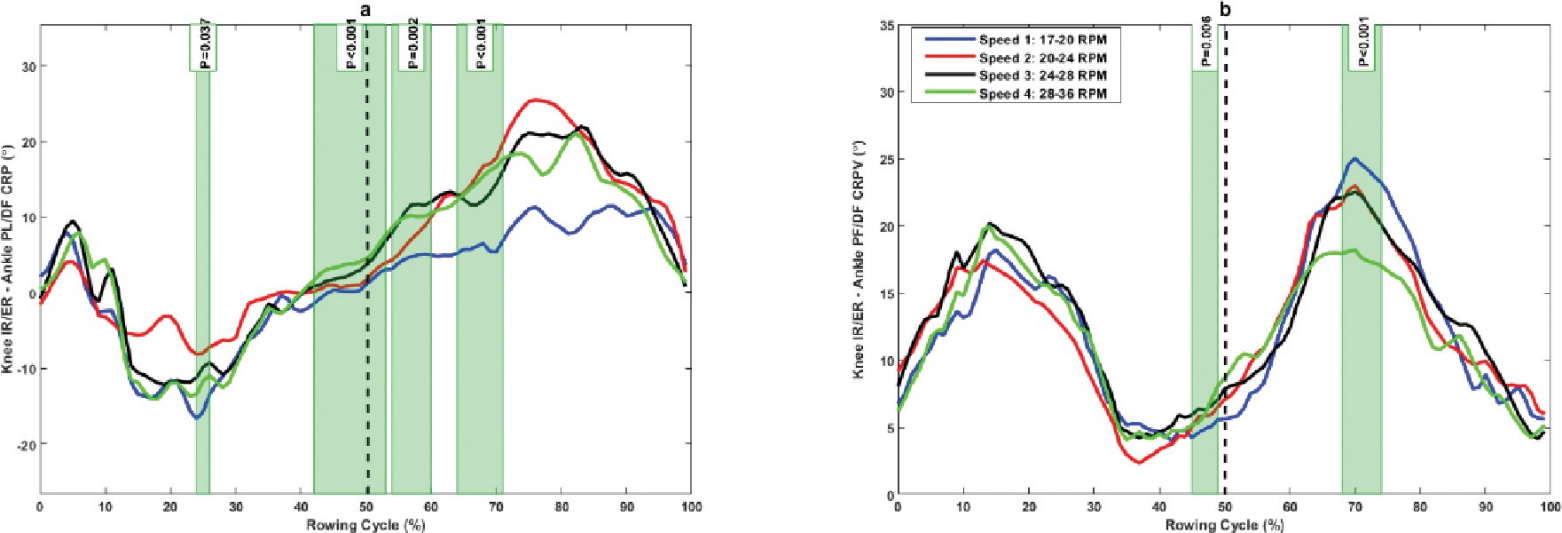
(a) Knee IR/ER–ankle PF/DF continuous relative phase and (b) its variability at different rowing speeds. The green shaded portions illustrate times in the stroke cycle when there was a significant difference between stroke rates.

The knee IR/ER–ankle PF/DF CRP was significantly less in-phase in the mid-drive phase (24–26%), but more in-phase in the late drive phase (42–50%), and early recovery phase (50%–56% and 63%–71%) (Fig 5A) at lower rowing speed compared to higher rowing speed. Moreover, rowers showed a significantly higher knee IR/ER–ankle PF/DF CRPV in the late drive phase (45%–49%), but less CRPV in the mid-recovery phase (68%–73%) at higher rowing speed compared to lower rowing speed (Fig 5B).

**Fig 6:**
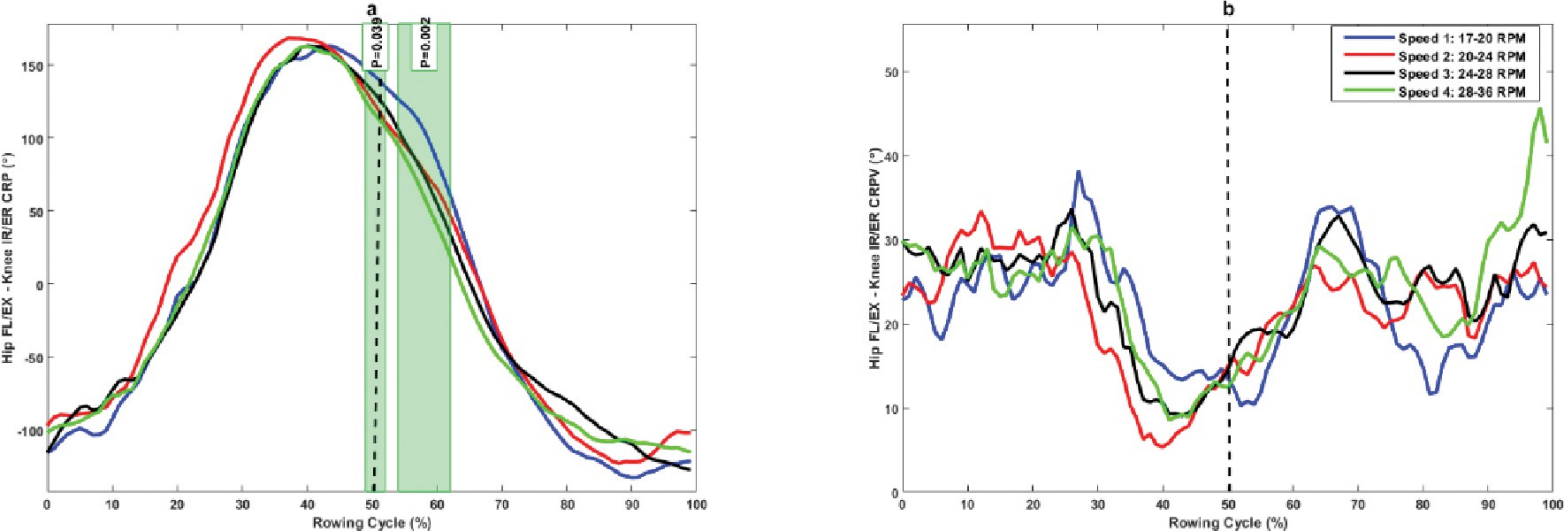
(a) Hip FL/EX–knee IR/ER continuous relative phase and (b) its variability at different rowing speeds. The green shaded portions illustrate times in the stroke cycle when there was a significant difference between stroke rates.

The hip FL/EX–knee IR/ER CRP was significantly more out of phase in the late drive phase (49%–50%) and early recovery phase (50%–51% and 53%–62%) at lower rowing speed compared to higher rowing speed (Fig 6A). However, rowing speeds did not exhibit any significant effect on hip IR/ER–ankle PF/DF VCRP during the rowing cycle (Fig 6B).

## Discussion

The purpose of this study was to examine the effects of rowing stroke rates on lower extremity inter-joint coordination and its variability in professional rowers. The results showed that joints coordination significantly differed at different rowing intensities in the rowing cycle, especially during the recovery phase, confirming our first hypothesis. The results also showed that rowers have an in-phase coupling pattern with ankle dominance in hip–ankle coupling. However, ankle dominance was reduced with an increase in rowing stroke rate, and this reduction was statistically significant at mid- and late drive phases and in the mid-recovery phase of rowing movements (Fig 2A). The hip–knee coupling pattern is also in-phase with knee dominancy, and it is very similar to the hip–ankle coupling pattern in the sagittal plane, and knee dominance was reduced with an increase in rowing stroke rate (Fig 4A). These results indicate that experienced rowers use more ankle plantar flexion and knee extension than hip extension for propulsion during the drive phase. This may be a dominant pattern for rowing task in experienced rowers, suggesting that rowers can rely more on ankle plantar flexors and knee extensors in drive phase of rowing. However, it need more researches to confirm this dominant pattern in experienced rowers. Furthermore, rowing with different coupling patterns in ankle, knee, and hip extension may increase the demand on hip and knee joints. This may not only affect their rowing performance but also increase the risk for overuse injury on proximal parts of the kinematic chain, as it has reported that inappropriate coordination patterns can cause stress in soft tissues, resulting in injury or discomfort [29, 30].

The results showed that ankle plantar flexion and knee extension dominancy were reduced and hip extension increased at higher rowing stroke rates, indicating that professional rowers change their motor strategies with increasing rowing intensity to increase their rowing speed. This may be a result of the force–velocity relationship: Rowers try to change their motor strategy and switch on their hip extensors to increase speed because of a decrease in the ankle and knee muscle force production at higher speeds. However, further electromyography analysis is needed to prove this idea. Most researchers have focused on the effects of rowing intensity on spine and pelvis kinematics as a result of the increased prevalence of lower back injury in rowers and our results cannot be compared with these studies directly [16, 18, 31–34]. Although they have not examined lower extremity joint coupling, our results align with those of Buckeridge et al. (2016), who have reported a reduction in ankle, knee, and hip ROM but an increase in L5/S1 ROM with an increase in rowing rate, which indicates an increase in joint ROM from distal to proximal of the kinematic chain with rowing intensity increasing [35].

The rowers exhibited an in-phase coordination pattern in the knee–ankle with knee dominance in the sagittal plane (Fig 3A). Knee dominance increased with increasing rowing stroke rate, but it was statistically different at the late drive phase and first half of the recovery phase of rowing movements. These results demonstrate the importance of knee extension in rowing tasks and increasing rowing speed during the drive phase of rowing. These results and the results of hip–ankle and hip–knee couplings reveal the importance of the knee joint in rowing tasks. This indicates the greater demands on the knee joint during rowing tasks and the increase in the possibility of knee overuse injuries. However, we did not investigate the coupling movement of the pelvis and spine in the kinematic chain of rowing movements. Because rowers use their pelvis and spine for trunk extension during the drive phase, future studies should investigate this segment and joint coupling in addition to lower extremity joints and segments to provide a better understanding of the role of joints in rowing tasks at different rowing speeds.

Experienced rowers exhibited an in-phase coordination pattern in knee IR/ER–ankle PF/DF with knee internal rotation dominancy during the drive phase of rowing and ankle dorsi flexion dominancy during the recovery phase of rowing. Moreover, the knee internal rotation and ankle dorsi flexion dominancy increased with increasing rowing stroke intensity so that knee internal rotation dominance was significantly greater at mid- and late drive phases and ankle dorsi flexion dominance was significantly greater during the early recovery phase of rowing (Fig 5A). More knee internal rotation during mid- and late drive phases, especially at higher stroke intensities, can increase the knee rotational loadings and may increase the risk for knee overuse and acute injuries. Moreover, the significant differences in this coupling during the late drive and early recovery phases indicate that the rowers use different motor strategies to transition from the drive to the recovery phase.

Experienced rowers exhibited in-phase coordination in hip FL/EX–knee IR/ER during mid-drive and mid-recovery phases, and there was an out-of-phase coordination in the remaining rowing phase (Fig 6A). However, there was just a significant increase in the out-of-phase coordination at greater rowing stroke rates during late drive and early recovery phases. The results indicate that there was hip extension dominance during the first half of the drive phase and also hip flexion dominancy during the second half of the recovery phase, and there was knee internal rotation dominancy during the second half of the drive phase and knee external rotation dominancy during the first half of the recovery phase. The knees extend fully during the last drive phase of the rowing task in a closed kinematic chain with a screw home mechanism. This means that the femur internally rotates to knee goes to close pack position at full knee extension [36]. This is a natural movement pattern of the knee joint; however, an increase in knee internal rotation dominancy can increase the demand on the knee joint, especially at higher rowing stroke rates, and may increase the risk for knee overuse and acute injuries.

Generally, the calculated coordination variability is reduced at higher rowing intensities, and in some rowing cycles it is significant, partially accepting our second hypothesis. It has been reported that increased variability of fluctuations is an essential feature of abrupt changes or phase transitions in movement patterns [37]. Moreover, reduction in effective degrees of freedom, interacting components, and synergies involved in the control of human movement may become associated with a loss of variability, and when these reductions in degrees of freedom and variability reach a critical threshold, injury or disease may emerge. The rowers showed a reduction in variability at the transmission of drive to recovery in all calculated couplings at all rowing intensities, which indicates a reduction in degrees of freedom (Figs 1B–6B). Thus, there may be a risk for injuries during transmission of drive to recovery as a result of reduction in coupling variability.

The rowers exhibited higher variability in hip–ankle coupling in the first half of the drive phase and generally showed a reduction in hip–ankle CRPV at higher rowing stroke, and it was significant at 27% of the drive phase (Fig 1B). A higher amount of variability was present in hip–knee coupling during the drive phase of rowing, but it was not statistically different among different rowing intensities. Moreover, the hip–knee coordination variability was greater but not statistically different among rowing intensities during the mid-drive phase of rowing, and this variability was significantly reduced in the mid-recovery phase with increasing rowing stroke rate (Fig 4B). The reduction in variability indicates a reduction in the degree of freedom at higher rowing strokes and changes in motor strategies in rowers to achieve higher rowing speed.

The results indicate that there was more variability in knee–ankle coupling in the sagittal plane during the first half of the drive phase, but it was significant between different intensities at 32% and 49% of the drive phase. Moreover, the knee–ankle coupling variability was significantly less at lower rowing intensities during the transition from the drive phase to the recovery phase (Fig 3B). This suggests that rowers have fewer degrees of freedom in this coupling at lower rowing intensities. There was greater knee IR/ER–ankle PF/DF coupling variability at mid-drive and mid-recovery phases of rowing, and this variability was significantly lower at higher rowing stroke intensity in the mid-recovery phase. Moreover, there was a significantly greater variability during the late drive phase at higher rowing stroke rates (Fig 5B). Researchers have reported that greater variability is an essential feature of abrupt changes or phase transitions in movement patterns [37]. The greater coupling variability in the mid-drive phase results from the ankle starting to move faster than the knee in phase space, and the greater coupling variability in mid recovery results from the knee starting to move faster than the ankle in phase space. The results indicate greater coupling variability in hip FL/EX–knee IR/ER in the first half of the drive phase and second half of the recovery phase of rowing (Fig 6B). This variability increased at higher rowing strokes during the late recovery phase, which is at the point of phase transition. However, this variability was not significant among different rowing stroke rates.

To the best of our knowledge, no previous studies have examined lower extremity coupling variability during rowing at different intensities, and we cannot compare the results with those of previous studies. In a single study in which the coupling variability between the spine and pelvis in healthy rowers in the sagittal plane [32] was analyzed, the authors suggested that thoracic–lumbar CV did not differ among different intensities but lumbar–pelvis CV significantly decreased in 80% intensity in the recovery–drive phase of rowing. The results of this study are roughly similar to those of Bailey et al. (2018), who reported significant differences in the VCRP of joints at different speeds [25]. However, our results contrast those of Abbasi et al. (2020) and Floria et al. (2019) for treadmill running because those studies did not report any differences in lower extremity coupling variability for changes in running speed [17, 38]. The examination of lower extremity segments and joints in the horizontal and frontal planes of movement in addition to the sagittal plane with other nonlinear calculations such as vector coding may provide a better understanding of coupling variability at different rowing stroke rates. Further research is needed to determine the effects of rowing speed on the coupling variability of the lower extremity. Finally, the participants of this research were young rowers and it may their potential segment length had effect on coupling results. Further research is needed to examine the lower extremity joints coordination patterns and its variability in matured professional rowers and compare their results with current research results.

## Conclusion

The results of this research demonstrate the importance of the knee joint in rowing tasks in professional rowers in submaximal rowing stroke rate and the shift of movement to the hip at higher rowing strokes. Moreover, the calculated coordination variability was greater at mid-drive and mid-recovery phases of rowing, but it was less during the transition from drive to recovery phases, and, overall, it decreased at higher rowing intensities. Further research is suggested to examine the lower extremity and pelvis and spine coupling variability at different rowing stroke rates to provide a better understanding of their role in rowing tasks at different speeds.
